# Vacuum sealing drainage combined with eggshell-like debridement antibiotic-loaded calcium sulphate for calcaneal osteomyelitis

**DOI:** 10.1186/s13018-023-04259-6

**Published:** 2023-10-24

**Authors:** Ruifang Yang, Haotian Hua, Xinwei Wang, Zairan Guo, Wenlong Zhong

**Affiliations:** 1 Department of Bone and Joint Infection, Luoyang Orthopedic-Traumatological Hospital of Henan Provincial, Luoyang, China; 2 Degree and Graduate Education Luoyang Work Department, Henan University of Traditional Chinese Medicine, Luoyang, China; 3 The First School of Clinical Medicine, Zhejiang Chinese Medical University, Hangzhou, China

**Keywords:** Eggshell-like debridement, Vacuum sealing drainage, Antibiotic-loaded calcium sulphate, Calcaneal osteomyelitis

## Abstract

**Background:**

To compare the clinical efficacy of vacuum sealing drainage, eggshell-like debridement combined with antibiotic calcium sulphate implantation and conventional debridement combined with antibiotic calcium sulphate implantation in the treatment of calcaneal osteomyelitis.

**Methods:**

Sixty-six patients with calcaneal osteomyelitis who were treated in our department between January 2017 and August 2021 were included in this study. Thirty-one patients underwent VSD and eggshell-like debridement combined with antibiotic calcium sulphate implantation. Thirty-five patients underwent conventional debridement combined with antibiotic calcium sulphate implantation. The inflammatory markers, operation time, wound healing time, hospital stay, full weight bearing time after operation, recurrence rate of infection, complications, and American Orthopedic Foot and Ankle Society (AOFAS) scores were compared between the two groups.

**Results:**

The operation time and full weight bearing time after operation of observation group were longer than that of control group. Compared with preoperative results, WBC, ESR, CRP and PCT in both groups were significantly decreased at 14 days after operation, and there was no statistical significance between the two groups. The wound healing time and hospital stay in the observation group were shorter than those in the control group (*P* < 0.05). There were four patients with aseptic exudation in the observation group and ten patients with aseptic exudation in the control group, and the wounds healed well after multiple dressing changes. Seven patients in the observation group underwent secondary bone grafting due to bone defects, and four patients in the control group received secondary bone grafting due to bone defects. In the observation group, three patients received debridement combined with antibiotic calcium sulphate implantation again due to recurrent infection, compared with seven patients in the control group. One year after operation, the observation group had a better AOFAS scores than the control group, especially in terms of foot function (*P* < 0.05).

**Conclusion:**

Compared with conventional debridement and antibiotic calcium sulphate implantation, VSD and eggshell-like debridement combined with antibiotic calcium sulphate implantation in the treatment of calcaneal osteomyelitis can shorten the wound healing and hospital stay of patients, reduce postoperative aseptic exudation complications and infection recurrence rate, and better preserve the foot function, which is a simple and effective method.

## Introduction

Calcaneal osteomyelitis is a common complication of open calcaneal fractures, open reduction fixation diabetes mellitus and hematogenous infections. The incidence of the disease accounts for approximately 3–11% of bone infections [1]. The special ‘skin-on-bone’ structure and biomechanical properties of the calcaneus can seriously affect walking function, with bone defects up to 13% [2]. This can make infection control difficult.

At present, the commonly treatment methods have problems such as high infection recurrence rate and disability rate [3, 4]. The eggshell-like debridement technique preserves the uninfected cortical bone of the calcaneus and removes the internal cancellous bone completely, balancing infection control with preservation calcaneal shape, facilitating postoperative calcaneal reconstruction and limbs function recovery [5]. In recent years, vacuum sealing drainage (VSD) and the antibiotic-loaded calcium sulphate have been used in the treatment of bone infections widely, with significant curative effects in filling bone defects, controlling bone infection and promoting wound healing [6, 7].

In this study, we reviewed sixty-six patients with calcaneal osteomyelitis and investigated the clinical efficacy of VSD combined with eggshell debridement and antibiotic-loaded calcium sulphate implantation in the treatment of calcaneal osteomyelitis compared with conventional calcaneal debridement and antibiotic-loaded calcium sulphate.

## Methods

Inclusion criteria: (1) Patients were treated at our hospital with a clear diagnosis of calcaneal osteomyelitis. (2) Surgical method with one of the two surgeries mentioned above. (3) The patient’s case information is complete and the follow-up is not lost. Exclusion criteria: (1) Patients have undergone partial or total calcaneal resection or ankle fusion previously. (2) Presence of a large soft tissue defect requiring flap treatment. (3) Patients with diabetic foot osteomyelitis or neuropathic diabetic foot ulcers. (4) Patients with cardiovascular, cerebrovascular, liver, kidney, blood system diseases. (5) Patients with poor compliance or who were not followed up.

From January 2017 to August 2021, a total of sixty-six patients who met the inclusion criteria were included in the study. Written informed consent was obtained from all patients. Patients were divided into observation group and control group according to different treatment. Observation group received VSD and eggshell debridement combined with antibiotic-loaded calcium sulphate implantation. The control group received conventional debridement combined with antibiotic-loaded calcium sulphate implantation. There was no significant difference in baseline data between the two groups. The information of the two groups of patients is shown in Tables 1 and 2.

**Table 1:** Descriptive data and clinical characteristics of patients

Variables	Observation group (*n* = 31)	Control group (*n* = 35)	*P* Value
Sex (Male/female)	24/7	26/9	0.77
Mean age (years)	45.32 ± 15.88	43.71 ± 14.90	0.67
Side			0.42
Left	12	17	
Right	19	18	
Cause of illness			0.49
Open wound	16	13	
Internal fixation of fractures	12	18	
Hematogenous	3	4	
Preoperative AOFAS score	59.61 ± 6.87	58.17 ± 6.79	0.40

**Table 2:** Comparison of bacterial culture results between the two groups of patients

Variables	Observation group (*n* = 31)	Control group (*n* = 35)	*P* Value
Bacteria positive and category(plants)			1.00
* Staphylococcus aureus*	6	6	
* Staphylococcus epidermidis*	3	4	
* Staphylococcus haemolyticus*	2	1	
* Pseudomonas aeruginosa*	4	5	
* Escherichia coli*	3	4	
* Enterobacter cloacae*	3	3	
* Klebsiella pneumoniae*	2	2	
* Acinetobacter baumannii*	2	2	
* Proteus mirabilis*	3	3	
* Corynebacterium*	1	2	
Culture negative (case)	5	7	0.68
Mixed infection (case)	3	4	0.82

### Surgical procedures

Preoperative treatment: After admission, all patients undergo X-rays of the calcaneus. Blood routine, erythrocyte sedimentation rate, C-reactive protein and procalcitonin were also tested. Bacterial culture was performed on patients with sinus passages, and sensitive antibiotics were selected based on the results of bacterial culture. Antibiotics were selected empirically for antibacterial treatment if the culture result is negative. The operation was performed when the patient was well.

**Fig. 1:**
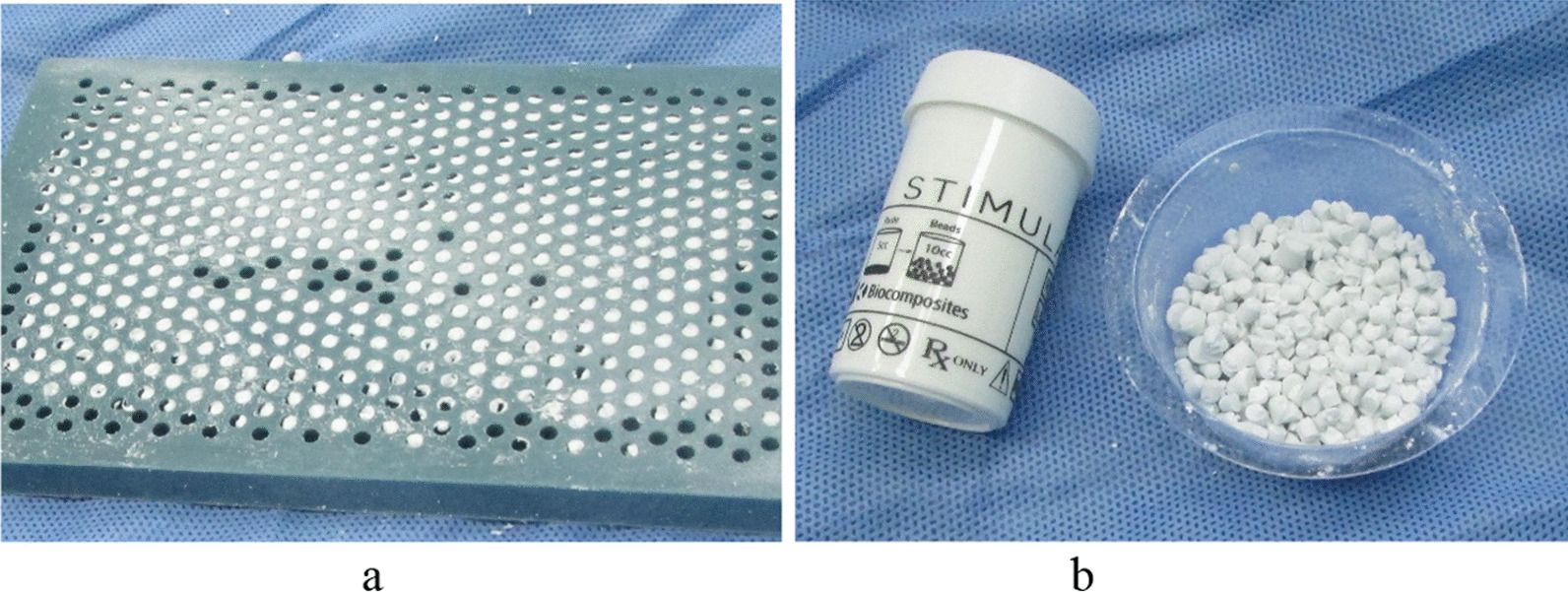
Preparation of antibiotic-loaded calcium sulphate pellets. **a** Paste calcium sulphate mixed with antibiotics is placed on a mould and left to dry and solidify. **b** Remove the solidified calcium sulphate pellets and set aside in a container

Surgical method: Methylene blue was injected into the wound or sinus after successful anaesthesia. The lesion was dissected along the original surgical incision or an L-shaped incision. Infected lesions should be exposed fully. If internal fixation exists, it should be removed first. During the operation, infected bones and soft tissues should be taken for bacterial culture and pathological examination. Depending on the extent of the methylene blue staining, the infected bone tissue and soft tissue were completely excised (The specific surgical method of debridement could be seen in the description of observation group and control group). After thorough debridement, the lesion was rinsed with normal saline and hydrogen peroxide three times, and then, soaked with iodophor solution for 10 min. Surgical towels, gloves and instruments were replaced. Vancomycin combined with gentamicin was selected for the treatment of gram-positive bacteria, and meropenem combined with gentamicin was selected for the treatment of gram-negative bacteria. The antibiotics were mixed with calcium sulphate powder and put into moulds to make granules (Fig. 1a, b). These particles were filled into the cavity formed by debridement. The drainage tube was placed, and the surgical incision was sutured.

Observation group: Bone knife or osteotomy device was used to remove infected and hardened necrotic cortical bone, leaving cancellous bone exposed clearly. Following the principle of “radical” debridement, the internal cancellous bone was scraped repeatedly with bone biters and curette. After debridement, the calcaneus took on an “eggshell-like” hollow structure. Drainage tubes were placed and surgical incisions are sutured. The foam dressing was trimmed according to the size of the surgical incision. The incision was covered with the foam dressing. The four corners were fixed with surgical sutures. VSD film was used to cover the foam dressing and the entire wound tightly. The drain tube was plugged into a negative pressure aspirator. The pressure is maintained at about 65 kPa.

Control group: The lesion was exposed fully. Locally infected necrotic cortical and cancellous bone was removed completely. In this process, the standard was following to remove the infected bone until healthy bone tissue reached spotting bleeding, which was called the “chili sign”.

Postoperative treatment: Routine anticoagulant and analgesic therapy was performed after operation. According to the results of preoperative bacterial culture, sensitive antibiotics were injected intravenously for 1–2 weeks and then, switched to oral antibiotics for 4–6 weeks. Three days after operation, patients were instructed to perform active and passive functional exercises. After the wound has healed fully, patients were allowed to load weight with crutch gradually. The time to complete loading should be determined by X-ray review results. In the observation group, patients were drained with VSD device for 7–10 days after operation. The colour and properties of the drainage fluid were observed. Fourteen days and seven days after operation, inflammatory markers are detected. After erythrocyte sedimentation rate (ESR), white blood cell (WBC), erythrocyte sedimentation rate (ESR), C-reaction protein, (CRP), procalcitonin (PCT) and other indicators decreased to normal, and no bacteria were cultured in the drainage fluid for three consecutive times, and the negative pressure drainage device was removed. In the control group, patients underwent regular surgical dressing changes, wound healing was observed, drainage tubes were kept unobstructed, and drainage tubes were removed at the right time. X-ray was performed regularly at 1, 3, 6 and 9 months after operation. If bone regeneration was poor, secondary bone grafting should be performed as soon as possible.

### Observation indicators

Inflammatory markers (including C-reactive protein, Erythrocyte sedimentation rate, White blood cells, Procalcitonin) were recorded. Operation time, wound healing time and hospital stay were recorded. Postoperative aseptic exudates, bone defects, and recurrence of infection have also been recorded. AOFAS scores were assessed at one-year follow-up after operation (The scoring criteria mainly include 40 points for pain, 50 points for foot function, and 10 points for alignment) [8].

### Statistical processing

The SPSS (Version 21.0) software package (SPSS Inc, USA) was used for statistical processing. Firstly, the normality and homogeneity of variance tests are performed on the measurement data. Measurement data conforming to normal distribution and homogeneity of variance were expressed as mean ± standard deviation (mean ± SD), and the two independent sample *T*-test was used to compare the differences between the groups. Measures that did not conform to the normal distribution and homogeneity of variance were expressed as median and interquartile range *M*(*P*_25_–*P*_75_), Wilcoxon rank sum test was used to compare the differences between groups. Counting data was expressed as frequency or percentage. Chi-square tests are used for comparisons between groups. *P* < 0.05 means there is a significant difference between the groups.

## Results

**Fig. 2:**
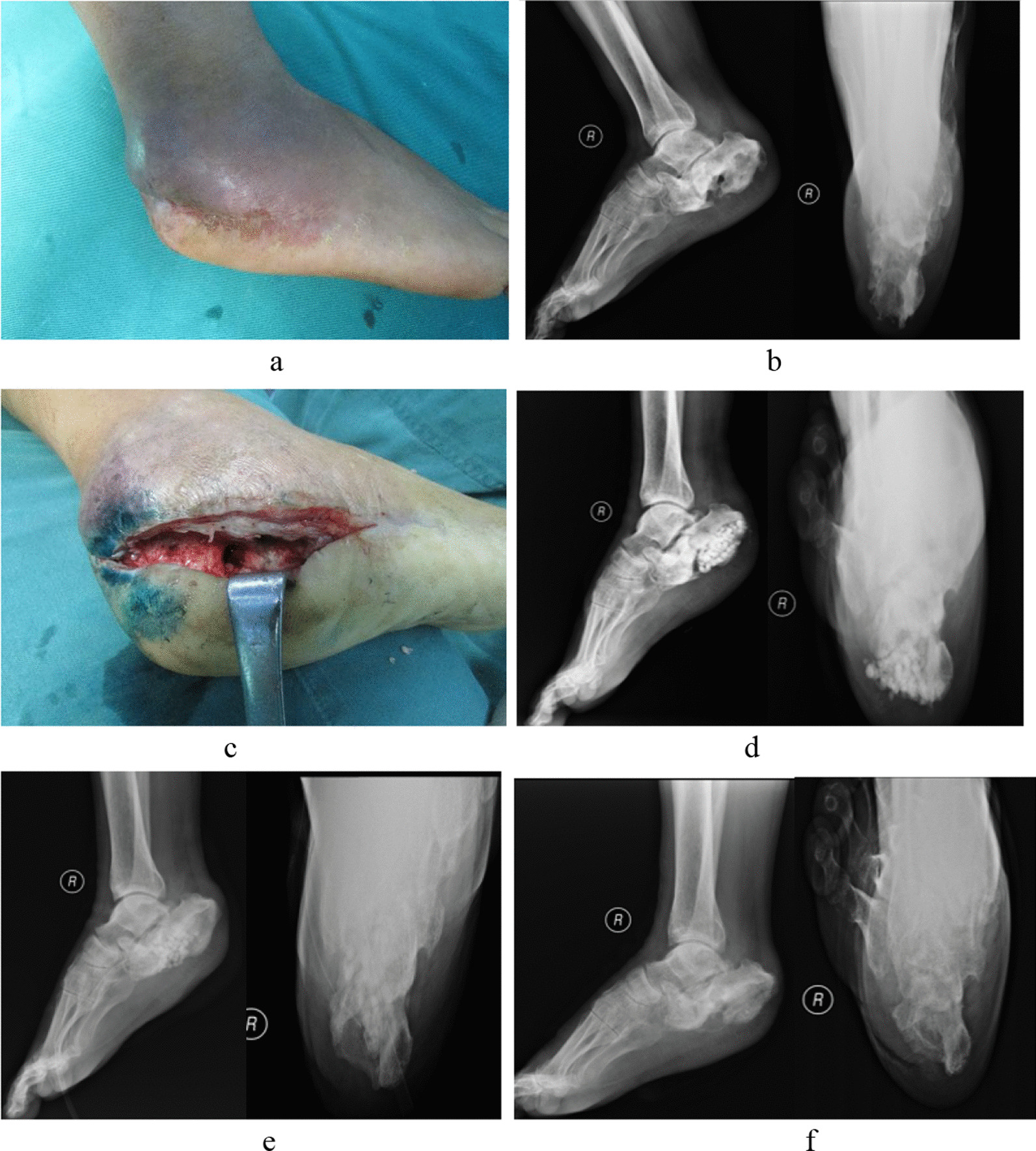
A 58-year-old man. Diagnosed with right calcaneal osteomyelitis. **a** Preoperative gross picture showed swelling and sinus formation in the calcaneal of the right foot. **b** Preoperative X-ray of the right calcaneal showed bone defect and sclerosis of the infected lesion. **c** Intraoperative methylene blue stained lesion is revealed. **d** One month after the operation, X-ray showed the implantation of calcium sulphate particles in the calcaneal bone. **e** Three months after operation, X-ray showed partial degradation of calcium sulphate; **f** One year after operation, X-ray showed new bone formation at the site of the defect

**Fig. 3:**
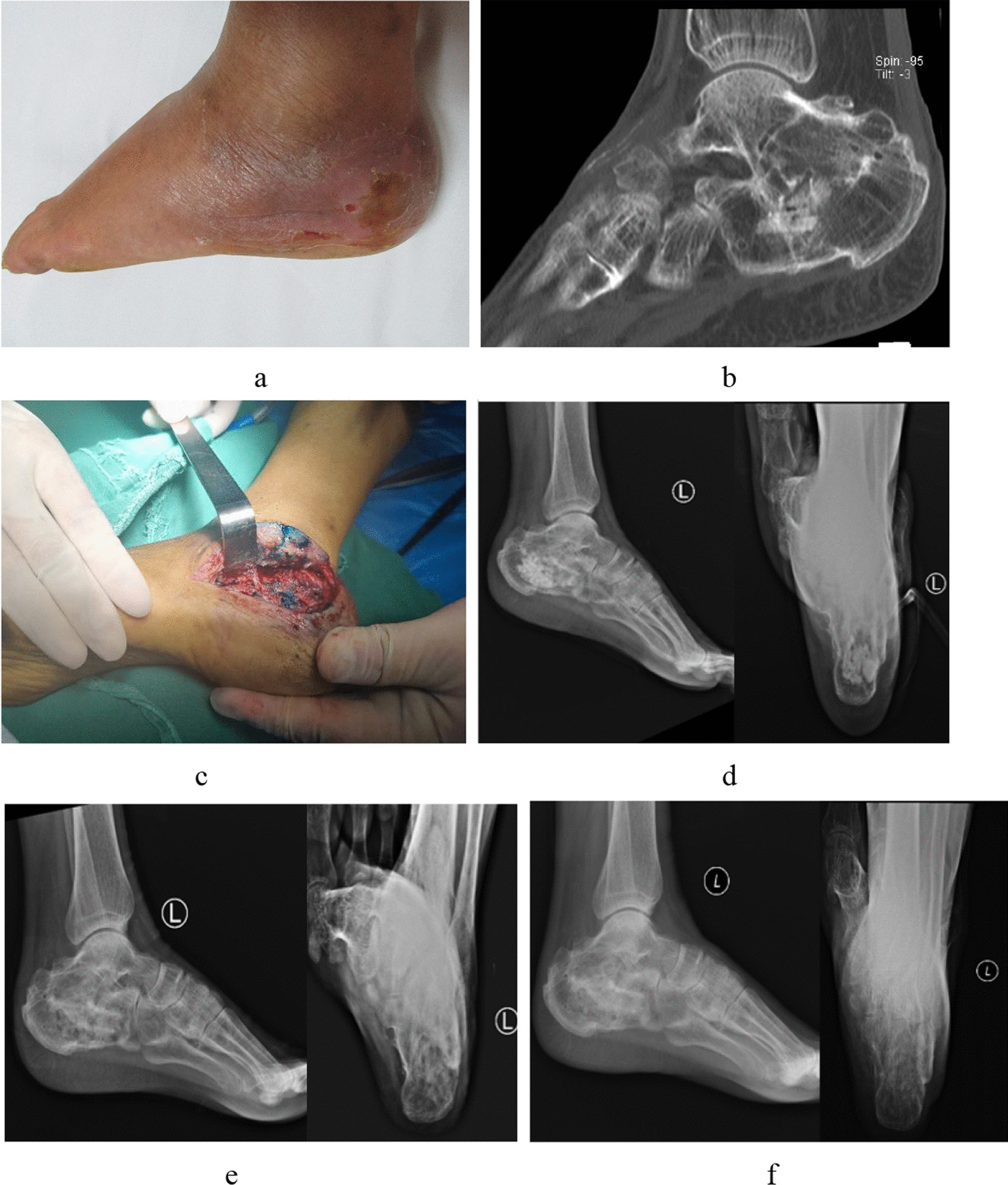
A 61-year-old man. Diagnosed with left calcaneal osteomyelitis. **a** Preoperative gross picture showed swelling and sinus formation in the calcaneal of the left foot. **b** Preoperative CT of the foot shows a local bone defect with multiple residual nail tracts. **c** Intraoperative methylene blue stained lesion is revealed. **d** One month after operation, X-ray showed the implantation of calcium sulphate particles in the calcaneal bone. **e** Three months after operation, X-ray showed calcium sulphate degradation, new bone formation and uneven increase in bone density. **f** Nine months after operation, the X-ray showed gradual uniformity of bone density and healing of the bone defect

The operation of all patients was completed successfully. The operation time and full weight bearing time after operation of observation group were longer than that of control group. The difference was significant statistically (*P* < 0.05). Wound healing time and hospital stay in the observation group were shorter than those in the control group (*P* < 0.05). Compared with preoperation, WBC, ESR, CRP and PCT were significantly reduced in the two groups at fourteen days after operation, but there was no significant difference between groups (*P* > 0.05). In the control group, two patients received secondary debridement and antibiotic calcium sulphate implantation due to poor infection control and non-healing wounds. The wound healed well after operation. All patients were followed up for 13–41 (average 26.61 ± 5.98) months after operation. There were four patients with aseptic exudation in the observation group and ten patients in the control group. The wound healed well after several dressing changes. Seven patients in the observation group received secondary bone grafting due to bone defects, and four patients in the control group received secondary bone grafting due to bone defects. In the observation group, three patients received the antibiotic calcium sulphate implantation again due to recurrence of infection. In the control group, seven patients received the antibiotic calcium sulphate implantation again due to recurrence of infection. The AOFAS score of the observation group was better than that of the control group 1 year after operation. Especially in function, the observation group was better than the control group significantly. Specific clinical results are shown in Table 3 (Figs. 2 and 3).

**Table 3:** Comparison of the postoperative conditions between the two groups of patients

Variables	Observation group (*n* = 31)	Control group (*n* = 35)	*P* Value
Operation time (minutes)	92 (78–92)	70 (64–70)	< 0.01
Wound healing time (days)	15 (13–16)	19 (15–24)	< 0.01
Hospital stay (days)	24.23 ± 4.18	31.51 ± 6.82	< 0.01
Postoperative full weight bearing time (weeks)	9.90 ± 2.07	8.14 ± 2.12	< 0.01
WBC (× 10^9^/L)			
Before operation	11.66 ± 3.61	11.31 ± 3.14	0.62
Fourteen days after operation	8.31 ± 1.94	8.50 ± 1.74	0.70
ESR (mm/h)			
Before operation	63.74 ± 26.37	64.34 ± 25.69	0.93
Fourteen days after operation	19.06 ± 7.89	21.06 ± 8.46	0.33
CRP (mmg/L)			
Before operation	56.6 (37–74.7)	54.6 (34.8–82.8)	0.85
Fourteen days after operation	6.4 (4.6–8.8)	9.4 (4.3–12.4)	0.25
PCT (ng/L)			
Before operation	0.75 ± 0.45	0.75 ± 0.42	1.00
Fourteen days after operation	0.25 ± 0.14	0.26 ± 0.15	0.83
The AOFAS score of one-year after operation	88.35 ± 6.81	83.86 ± 8.90	0.03
Pain	40 (30–40)	40 (30–40)	0.27
Function	43.84 ± 3.60	41.54 ± 4.00	0.02
Alignment	5 (5–10)	5 (5–10)	0.69

*WBC* white blood cell, *ESR* Erythrocyte sedimentation rate, *CRP* C-reactive protein, *PCT* procalcitonin

## Discussion

### Advantages of eggshell-like debridement techniques for calcaneal osteomyelitis

Thorough debridement of the infected lesion is the key to the treatment of osteomyelitis. The calcaneus is an important weight-bearing bone of the human body, and it is also an irregular tarsal bone, which is not fully applicable to the Cierny–Mader classification criteria and debridement method based on long bone infection [9, 10]. The Gaenslen technique, partial or complete calcaneal resection, and subknee amputation are currently commonly used methods of calcaneal debridement, but there are disadvantages of incomplete debridement, recurrence of infection, and damage to the shape and function of the calcaneus [11, 12].

The eggshell-like technique was proposed by Heinig et al. in the 1970s and was used for debridement and orthopaedic treatment of vertebral infections. The technique is named for the eggshell-like shape of the preserved cortical bone [13]. The anatomy of the calcaneus is similar to the vertebrae, and the eggshell-like debridement technique was first used for calcaneal osteomyelitis by Cheng-he Qin et al. [5]. Postoperative foot function was preserved, and painless weight bearing was achieved in most patients. Compared to conventional debridement, eggshell-like debridement has the following advantages: (1) The irregular tarsal characteristics and weight bearing function of calcaneal bone were fully considered in eggshell debridement. The infected cortical bone site is opened, the internal cancellous bone is scraped, and the uninfected cortical bone is preserved to maintain the shape of the calcaneus, which is conducive to postoperative calcaneal reconstruction and foot function recovery. In this study, it was also found that the AOFAS score, foot function of the eggshell-like debridement group were better than those in the conventional debridement group (*P* < 0.05) 1 year after operation, and the shape and function of the calcaneus were fully preserved. (2) The superficial cortical bone of calcaneus contains a large amount of spongy cancellous bone, which has loose trabeculae, numerous pores and slow blood flow rate, making it a breeding ground for bacteria to grow and proliferate. Even experienced clinicians are difficult to clearly define the scope of infection and the degree of debridement, resulting in a high recurrence rate of calcaneal infection after debridement. The eggshell-like debridement removes as much cancellous bone as possible from the cortical bone, making debridement more complete and reducing the potential for recurrence of postoperative infection. In this study, the infection recurrence rate in the eggshell-like debridement group was lower than that in the conventional debridement group. However, it is worth noting that eggshell-like debridement removes cancellous bone as much as possible, not only does the operation and full weight bearing take a long time, but there is a problem of bone regeneration, and a second stage of bone grafting is required to reconstruct the calcaneus. In short, eggshell-like debridement is an effective debridement method for calcaneal osteomyelitis with the dual goals of controlling infection and preserving the shape and function of the calcaneus.

### Therapeutic advantages of VSD in combination with the antibiotic-loaded calcium sulphate implantation

The application of sensitive antibiotics, elimination of the cavities and adequate drainage are the basic principles in the treatment of bone infection [14]. Antibiotic-loaded calcium sulphate implantation can avoid bacterial resistance and drug toxicity caused by long-term systemic application of antibiotics, rapidly and stably release antibiotics at high local concentrations, and can induce bone regeneration [15, 16]. The combination of the antibiotic calcium sulphate implantation and VSD has the following advantages [17]: (1) Antibiotic calcium sulphate implantation can fill the cavity caused by debridement, continuously release antibiotics at high concentrations to kill local bacteria and can also induce bone regeneration, accelerate calcaneal reconstruction, and provide mechanical support for the calcaneus, allowing patients to bear weight early [18]. (2) The skin around the calcaneus has poor elasticity and high tension. The vacuum negative pressure generated by VSD foam dressings can exert mechanical force on wounds, which can reduce local tissue oedema and soft tissue tension of the calcaneus. Studies have shown that mechanical stimulation plays an important role in regulating the polarization process of macrophages, which dominates wound healing [19]. This study also found that the wound healing time and hospital stay in the observation group were significantly shorter than those in the control group, which helped accelerate the recovery of patients and reduce the financial burden. (3) VSD can close tissue spaces after debridement, reduce blood and fluid accumulation, and continuously and fully drain infected necrotic tissue and calcium sulphate metabolites, which will effectively reduce aseptic exudation complications after calcium sulphate implantation and infection recurrence. In this study, the rates of aseptic exudate and recurrence of infection in the observation group were lower than those in the control group and previous related studies [4, 5]. The combination of VSD and antibiotic-loaded calcium sulphate, following the principles of bone infection treatment of local antibiotic application, elimination of cavities and adequate drainage. This method can effectively control calcaneal infection, accelerate wound healing, shorten hospital stay, reduce postoperative infection recurrence and postoperative aseptic exudation.

### Considerations for VSD combined with eggshell-like debridement antibiotic-loaded calcium sulphate implantation

In the treatment of calcaneal osteomyelitis using VSD and eggshell-like debridement combined antibiotic-loaded calcium sulphate implantation, the following issues need to be noted: (1) The application site and duration of VSD are still controversial [20]. We usually place the VSD foam on the surface of the tightly stitched surgical incision rather than inside the incision, which avoids accelerated elution of antibiotic calcium sulphate and maintains local antibiotic concentration. The "honeycomb" structure of VSD foam is a reservoir for microorganisms, and studies have shown that the duration of foam retention is an independent risk factor for recurrence of infection [21]. Therefore, the VSD application time should not be too long. We think 7–10 days is the right time. If the time needs to be extended, the VSD should be replaced in time. (2) Postoperative care of VSD needs to be paid attention to. Drainage needs to be kept unobstructed to avoid ineffective drainage such as blood accumulation, air leakage and water leakage, especially the aseptic exudate produced by calcium sulphate metabolism, which is easy to block the drainage tube. (3) The choice of antibiotic is based on the type of bacterial culture. Vancomycin in combination with gentamicin is used by us to treat gram-positive bacteria, and meropenem in combination with gentamicin is used by us to treat gram-negative bacteria. For patients without cultured bacteria, vancomycin and gentamicin were selected. Calcium sulphate powder should be prepared into cylindrical particles with a diameter of 3–5 mm to increase the contact area with the lesion, which can accelerate the release and degradation of antibiotics and control infection better. (4) Calcium sulphate produces a lot of liquid as it degrades. Clinicians need to inform patients in advance to avoid causing concern. (5) The eggshell-like debridement only preserves part of the cortical bone. The mechanical strength of the calcium sulphate is poor, so it is necessary to delay the weight-bearing time. The time of full weight-bearing should be determined based on the results of the calcaneal X-ray to prevent pathological fracture. The calcaneal X-ray should be reviewed regularly after operation to assess the infection control and bone regeneration. If bone regeneration is poor, secondary bone grafting should be performed as early as possible to reconstruct the calcaneus.

In conclusion, compared with conventional debridement antibiotic calcium sulphate implantation, eggshell-like debridement combined with VSD and antibiotic-loaded calcium sulphate implantation can shorten wound healing and hospital stay, reduce postoperative aseptic exudation complications and infection recurrence rate, and preserve foot function better, which is a simple and effective method. However, this method also has some limitations in clinical application. For patients with calcaneus or soft tissue defects, bone and soft tissue reconstruction with flap or bone graft is required in the second stage. In addition, the cavity after eggshell-like debridement of calcaneus is large, and the mechanical strength of implanted calcium sulphate is poor. As an important weight bearing bone, the calcaneus is prone to pathological fractures due to improper weight bearing after operation. With the development of tissue engineering in the future, it is hoped that the emergence of new antibiotic-loaded bioactive materials can overcome the above drawbacks.

## Data Availability

All authors and institutions can access the raw data by directly contacting the author at 317940658@qq.com by reasonable request.

## Abbreviations

AOFAS: American Orthopedic Foot and Ankle Society
VSD: Vacuum sealing drainage
WBC: White blood cell
ESR: Erythrocyte sedimentation rate
CRP: C-reactive protein
PCT: Procalcitonin

## References

[1] McCann MJ, Wells A (2020). Calcaneal osteomyelitis: current treatment concepts. Int J Low Extrem Wounds.

[2] Oliver NG, Steinberg JS, Powers K (2015). Lower extremity function following partial calcanectomy in high-risk limb salvage patients. J Diabetes Res.

[3] Zhang L, Chen YS, Wang XW (2021). Simultaneous treatment of traumatic calcaneal osteomyelitis and defect deformity with near-arc bone transport by Ilizarov technique. Chin J Repar Reconstr Surg.

[4] Jiang N, Zhao XQ, Wang L (2020). Single-stage debridement with implantation of antibiotic-loaded calcium sulphate in 34 cases of localized calcaneal osteomyelitis. Acta Orthop.

[5] Qin CH, Zhou CH, Ren Y (2020). Extensive eggshell-like debridement technique plus antibiotic-loaded calcium sulphate for one-stage treatment of chronic calcaneal osteomyelitis. Foot Ankle Surg.

[6] Badie AA, Arafa MS (2019). One-stage surgery for adult chronic osteomyelitis: concomitant use of antibiotic-loaded calcium sulphate and bone marrow aspirate. Int Orthop.

[7] Luo J, Niu RF, Lv LF (2022). Application of vancomycin-loaded bone cement combined with vacuum sealing drainage in moderate to severe diabetic foot infection. J Chongqing Med Univ.

[8] Schneider W, Jurenitsch S (2016). Normative data for the American Orthopedic Foot and Ankle Society ankle-hindfoot, midfoot, hallux and lesser toes clinical rating system. Int Orthop.

[9] Cierny GR, Mader JT, Penninck JJ (2003). A clinical staging system for adult osteomyelitis. Clin Orthop Relat Res.

[10] Zhou CH, Management of diabetic forefoot osteomyelitis and chronic calcaneal osteomyelitis with antibiotic-loaded calcium sulfate. Southern Medical University, 2020.

[11] Bhattacharyya A, Jha AK, Kumar S (2012). Outcome of different modalities of surgical management of chronic osteomyelitis of calcaneum. J Indian Med Assoc.

[12] Zhao XL, The Clinical curative effect observation of the extent of calcaneal resection to treat the Calcaneal osteomyelitis. Shandong University of Traditional Chinese Medicine, 2012.

[13] Du HY, Liu J, Guo ZJ (2017). Clinical study on the application of Masquelet technique in the treatment of calcaneal osteomyelitis. Orthop J China.

[14] Xie Z (2018). Reflections on bottlenecks in bone infection control. Chin J Orthop.

[15] Cobb LH, McCabe EM, Priddy LB (2020). Therapeutics and delivery vehicles for local treatment of osteomyelitis. J Orthop Res.

[16] Jin ZC, Cai QB, Zeng ZK (2018). Research progress on induced membrane technique for the treatment of segmental bone defect. China J Orthop Traumatol.

[17] Haidari S, Ijpma F, Metsemakers WJ (2021). The role of negative-pressure wound therapy in patients with fracture-related infection: a systematic review and critical appraisal. Biomed Res Int.

[18] Li Q, Song SF, Zhang W (2017). Clinical study on negative pressure closed dr rainage combined with vancomycin loaded calcium sulfate and autogenous bone in the treatment of chronic osteomyelitis. China J Orthop Traumatol.

[19] Xu CL, Yu D, Zhu HY (2022). Research progress on the regulation of macrophage polarization by mechanical stimulation in wound healing. Chin J Reparat Reconstr Surg.

[20] De Feo M, Vicchio M, Sante P (2011). Evolution in the treatment of mediastinitis: single-center experience. Asian Cardiovasc Thorac Ann.

[21] Sweere V, Sliepen J, Haidari S (2022). Use of negative pressure wound therapy in patients with fracture-related infection more than doubles the risk of recurrence. Injury.

